# History of the Symptoms of J. P. F., Previous to Death, by Letter from Dr. Clark; Also, Synopsis of Notes Taken at the Post Mortem

**Published:** 1864-07

**Authors:** Hiram Wanzer

**Affiliations:** Chicago, Ill.


ARTICLE XXIX.

### HISTORY OF THE SYMPTOMS OF DR. J. P. F. PREVIOUS TO DEATH,

### BY LETTER FROM DR. CLARK;

### ALSO, SYNOPSIS OF NOTES TAKEN AT THE *POST MORTEM.*

By HIRAM WANZER, M.D., of Chicago, Ill.

Communicated to the Chicago Medical Society. May 20, 1864.

Dr. John S. Clark states as follows:—Was consulted by a friend of Dr. Jacob P. Fuller, on the 9th May, who thought he was becoming insane. From the description of the case I thought so too, and advised his removal to the asylum.

Two or three days after, I saw him in the street. My attention was drawn to him by his nervous and excited manner. On Sunday, the 15th, was called to see him, about 1 o'clock P.M. Found him lying upon a sofa; head on pillows; and breathing peculiarly. Some half dozen short, light, easy respirations, and then a deep one, with a noise as of strangling; his lips were livid; pupils contracted; pulse 80 and regular. He was in a state of profound stupor. Had him placed in the middle of the room, with his head and shoulders elevated, and all the windows and doors open; dashed his head with cold water, and covered his legs and feet with mustard plasters. Fearing some narcotic poison, tried to use the stomach-pump, but could not, owing to the tube, which was old, and softened as soon as warm, collapsing. Then gave an emetic of sulphate of zinc, which took half an hour to give, for fear of strangling. All this time his feet and legs were bitten by the mustard; his hands and arms were thoroughly rubbed and slapped; the emetic did not operate. The stupor continued; and from the beginning to the end of the case, there was not the least sign of consciousness. The respirations became more difficult and often ceased altogether, for an interval of a minute or so, then rallying with a great gasp. At such times the pulse would falter, and fall as low as 36, about five hours after being taken; large quantities of pink-colored frothy mucus was forced out of the mouth and nostrils, and continued for over an hour to flow quite freely; at this time the pulse at the wrist had ceased.

At 8 o'clock he died. J. S. CLARK, M.D.

*No.* 333 *North Wells Street.*

MR. PRESIDENT AND GENTLEMEN:—It was my privilege, on Monday evening, this week, to assist Dr. HATCH at a *post mortem* upon the body of Dr. J. P. F., dentist, aged 24. Autopsy revealed death from cerebral apoplexy or hæmorrhage of the brain. No serous effusions were found upon removing the calvarium; no external lesions, ecchymosis, or abrasions whatever were found to indicate the cause of death from outward violence. The dura mater was apparently normal; the arachnoid and pia mater were adherent closely to it by previous plastic inflammation. The cerebral surface of the pia mater was covered with a layer of sizy blood, indicating there had been at one time active meningitis. The second pathological condition discovered was a quantity of coagulated blood around the optic nerves, optic commissures, optic tracts, which in my opinion did account for paroxysmal pains over the orbits, frequently amounting to agony. During such periods, he would throw the hand to the forehead and with its palm make pressure at that point. There were clots at different points in both cerebral hemispheres all that we discovered. There was especially one large clot extending over the floor of the right lateral ventricle, covering part of the velum interpositum, thalami optici, and corpora striata. The blood might have proceeded from the venæ galeni, choroid plexus, and veins of the corporis striata. The various clots throughout the cerebral hemispheres assumed different colors, indicating those lesions were not all recent. The cerebral substance was nearly unaltered in color, save at those points of extravasation, and I think generally whiter than normal and softer in consistence. The exceeding hurry and lateness of the hour forbid our making as thorough an examination as we desired; for I think the microscope would have revealed changes in the arterial and venous coats, perhaps fatty degeneration of their texture, and pus globules in their channels, and other pathological changes in the cerebral texture our natural eyes did not nor could not detect.

It was our opinion that there were found in the brain pathological changes sufficient to cause death, without examining further other organs essential to life. That those blood clots imprisoned within the cerebral substance had so interrupted the functions of the brain as to cause those symptoms and his early and much lamented death. For several weeks previous to his dissolution, his friends noticed at times peculiar signs of delirium. One habit he acquired of constant expectoration of frothy, colorless mucus; also, occasional incoherent talking. He had frequent forebodings and hours of mental despondency. His mind wandered frequently upon unreal objects, often apprehensive of his professional friends, imagining they were trying to destroy a professional existence he was struggling to establish. At times he apprehended his dearest friends were mixing poison with his food, and it is said he once went to Detroit for his dinner to avoid being poisoned. He occasionally refused all nourishment except from the hands of one very dear and valuable friend in whom his confidence was only separated by death. He suffered frequent tingling prickling sensations in the extremities, significant of approaching paralysis. There was a peculiarity noticed in his walk—his head bowed and eyebrows knit as though he was in deep meditation. At times he was very irritable and irascible in temper. This irritability always appeared beyond his control. The carotids and temporal arteries would then beat vehemently. The vessels of the head and face would become suddenly injected—the skin of a firey red color.

Might not a series of those paroxysms of morbid excitement, causing such rapid determination of blood to the brain, have induced rupture of attenuated and diseased bloodvessels, causing those extravasations within the cerebeal substance, at various periods. As from the appearance of the blood clots some were old, others recent, coupled with an abiding sub-acute meningeal inflammation, causing the effusion over the cerebral surface of the pia mater. The opinion of the Society is solicited.

